# Supplementary material for: The spatially heterogeneous and double-edged effect of the built environment on commuting distance: Home-based and work-based perspectives
Source: PLoS One. 2022 Mar 23;17(3):e0262727. doi: 10.1371/journal.pone.0262727 (PMC8942273; doi:10.1371/journal.pone.0262727)
Supplement: Attachment — Submitted filename: Response to reviewers1216.docx [file pone.0262727.s002.docx]

GENERAL COMMENTS TO THE EDITOR AND REVIEWERS:

We are pleased to resubmit our revised manuscript of PONE-D-20-19283, which entitles ‘Spatially heterogeneous and double-edged effect of built environments on aggregated home-based and work-based commuting’. We appreciate thoughtful comments and constructive suggestions of the reviewers.

We explain how we revised the paper based on each comment as outlined below.

RESPONSE TO REVIEWER #1:

This paper aims to examine the spatially heterogeneous impact of built environment on commuting distances using mobile phone data in Guangzhou, China. Some questions need to be addressed before consideration of publication:

1. The Literature review section needs to be reorganized. The two subsections have both reviewed the impacts of built environment on commuting. The spatial heterogeneity is not clear in the review section.

Response: Thanks for your comments. We totally rewrote the literature review part (lines 129-235). The new one has reviewed three aspects of spatial heterogeneity analysis: differences between centers and suburbs, a multi-level model, and a GWR model. It enhances the theoretical construction of this study according to your suggestion.

2. What is the difference between home-based and work-based commuting? Please make it clearer. In many cases, they might be similar. Commuting is influenced by built environment at both residential location and workplace.

Response: Thanks for your suggestion. Home-based and work-based commuting are differentiated by two groups of commuters – residents and employees - in the same spatial unit. They are not differentiated by land-use of residence and work. For example, departure trips in the morning in a work-rich area is home-based commuting because we are observing commuters who live there. A same built environment variable’s impact is on two groups of people, that’s why we separate them and propose the ‘double-edged effect’ hypothesis. We gave a more details that:

**‘It is important to note that, for the same spatial unit or neighborhood, there might be two distinct groups of commuters with totally different behavior: residents and employees. The former is home-based commuters who depart from the spatial unit, while the latter is work-based commuters who arrive at the spatial unit. Because of the behavioral differences of two commuter groups, it is necessary to differentiate between home-based and work-based commuting. ‘(lines 79-85).**

**‘In this study, an underlying hypothesis is that the built environment’s impact on home-based and work-based commuting may be different or even contrary, causing the double-edged effect. To examine the double-edged effect, it is necessary to analyze the relation of commuting and built environment from both home-based and work-based perspectives.’ (lines 91-96)**

3. Some figures are in poor quality, please modify them. Moreover, it had better provide some figures to illustrate the spatial distribution of built environment in Guangzhou before running the model.

Response: Yes, we have improved quality of figures. The poor figure quality may be due to the compression of figures in the submission system. Original figures can be downloaded from the link at right upper corner of figure pages. We also provide the spatial distribution of built environment variables in Fig 2.

4. Please give more details on the data.

Response: We gave more details on the cellphone data and POI data. For example, we added in lines 254-260 that **‘The cellphone data have identified 13.7 million commuting trips in one month.’ And that ‘Points of interest (POIs) data are also applied to represent the built environment. A piece of ‘point of interest’ data records information of a coordinate location and a functional type of a spatial facility from a navigation map. POIs data are sourced from the Baidu map and provided by the Daodaotong company. Totally 27349 pieces of POIs are used in this study.’**

We also give details of recreation and transport POIs in variable description part (lines 312-315) that **‘Recreation POIs include dining (20.6%), public service (15.7%), entertainment (11.5%) and shopping (52.2%). Transport POIs include the parking logs (51.6%), car services (34.0%), and important transport navigation spots such as toll gates, bridges, train/bus stations, etc. (14.4%).’**

5. There are several grammar errors in the paper. Please improve the language.

Response: Yes, we did another round of proofreading throughout the article. We have revised more than 200 grammar errors or inappropriate sentences.

RESPONSE TO REVIEWER #2:

The paper uses geographically weighted regression models to examine the spatially heterogeneous effect of the built environment on commute. The authors concluded that the impact from built environment might vary over space and also be different for home-based and work-based commuting. I have several major concerns/suggestions:

• The contribution of the paper is weak. The authors need to highlight the values of your paper. If the value is to examine the spatial heterogeneity in the relationships, literature review needs to be rewritten with the focus of spatial context. What are the study areas of the existing studies? Is it region-wide, city-wide, or country-wide study? Could the complicating results be due to differences in spatial context? Also, need to add a conceptual framework explaining why the relationships could be spatially varied. Why separating home-based and work-based commutes and why the effects could be double-edged? Your conceptual framework should built upon existing studies.

Response: Thanks for your thoughtful suggestions. In this round of edit, we made a major revision on the literature review part. We totally rewrote it and emphasized the spatial heterogeneity. Related studies are reviewed by three aspects: differences between centers and suburbs, a multi-level model, and a GWR model. See contexts in lines 130-235.

Commuting is basically a city-wide issue, although some studies compare different cities’ commuting and built environment relations. This study narrows it in a city-wide spatial context. We state it in contexts **‘there are still controversies on how they affect commuting in a city-wide spatial context (lines 46-47)’** and **‘taking a city or a part of a city as cases, studies of the heterogeneous impact of the built environment can be summarized in three aspects. (lines 140-142).’** Exploring the spatial heterogeneity in a city-wide improves our understanding of the urban space in a finer scale that **‘Since commuting is generally a city-wide issue, the heterogeneous effect should be revealed explicitly on a finer spatial scale. Therefore, it is necessary to apply a local model, a geographically weighted regression model e.g., to give an overview of the structural relation between commuting and the built environment of the whole city. (lines 230-233)’**

Separating home-based and work-based commuting is because they represent different behavior of two commuter groups, those depart from a spatial unit and those arrive at a spatial unit. A same built environment variable’s impact is on two groups of people, that’s why we separate them and propose the ‘double-edged effect’ hypothesis. We gave more details:

**It is important to note that, for the same spatial unit or neighborhood, there might be two distinct groups of commuters with totally different behavior: residents and employees. The former is home-based commuters who depart from the spatial unit, while the latter is work-based commuters who arrive at the spatial unit. Because of the behavioral differences of two commuter groups, it is necessary to differentiate between home-based and work-based commuting. (lines 79-85).**

We also added a conceptual framework in Fig 1, and the article is re-organized by answering four research questions according to the framework.

• “Dependent variable is decay parameter beta in Eq.1. It represents the mean of the distance distribution.” Is it the mean of the distance or the slope of the decay? Please explain why using beta as dependent variable and refine your interpretation of beta. If it represents the mean, why not simply using the mean of all local distances? Your result interpretation need to be consistent with your definition of beta. For example, on page 9, “Residential population increases commuting distance….” It should be residential population increases beta, correct?

Response: Thanks for your comment. Please see our response as follows: ‘**The decay parameter also represents the expectation of the distance distribution in an exponential distribution.** $\boldsymbol{\beta}$ **is not simply a mean of commuting distances. Rather, we should treat it as a weighted average of that, according to the property of an exponential distribution. In other words, the expectation value also shapes the slope of the curve. It represents either a high probability of longer distances and a low probability of shorter distances (large** $\boldsymbol{\beta}$**), or a high probability of shorter distances and a low probability of longer distances (small** $\boldsymbol{\beta}$**). The decay parameters** $\boldsymbol{\beta}$ **of commuting distance distributions are differentiated by departure trips and arrival trips, which represent home-based and work-based commuting respectively. (lines 283-291)’**

• The independent variables need clarifications. How are independent variables selected? For instance, why commercial and recreation function could influence commute? Are the built environment variables measured for a single cell? Are surrounding cells included? “Infrastructure POIs represent the transportation facilities”. What types of transportation facilities? Why Closeness and Distance to center are global variables?

Response: Thanks for your questions. We answered them in our manuscript: **Independent variables (Table 1) are selected according to ‘Ds’ measurement [42] of the built environment, including density, diversity, design, destination accessibility, etc. (lines 292-293)**

Influences from commercial and recreation function on commuting can refer to previous studies. They are explained in lines 304-309.

**All variables are measured in a single cell without including surrounding cells. Since the GWR model itself is a spatially weighted algorithm considering the built environment’s impact from surrounding spatial units, there is no need to measure the built environment from adjacent cells again. (see lines 343-347)**

**Transport POIs include the parking logs (51.6%), car services (34.0%), and important transport navigation spots such as toll gates, bridges, train/bus stations, etc. (14.4%). Transport facilities are mainly associated with vehicle trips. (see lines 314-315)**

**Built environment variables should be further defined as local ones and global ones in a GWR Model. A local variable means its coefficient value varies across different locations, while a global variable has a unique coefficient value across the entire study area like a linear regression model. Variables not capable to capture the spatial relation, such as residential population, work population, recreation POIs, transportation POIs, and POI mixture, are local variables. The value of local variables varies across different locations. Variables of closeness and distance to the center, differently, are global variables since they represent the location effect. These variables themselves can describe spatial relation to the city center. (lines 335-343)**

• The current study area is the inner city of Guangzhou? Any particular reasons why the paper only focus on the inner city?

Response: Actually the study area is the urbanized area of Guangzhou. We explained it in texts that ‘**the study area is the urbanized area but not the whole city. The urbanized area of the city has an area of 2435.7 km^2^ and a population of 11.5 million in 2019^[1]^. The city’s population is concentrated in the urbanized area that 75% of the population is distributed at 32% area. The rest un-urbanized area is mainly rural and forest land with sparsely distributed residential settlements. That is the reason why we select the urbanized area as the study case. (lines 239-244)’**

• Please also add more discussions about the value of the cluster analysis. Within a single cluster, a variable could also have double-edged effect on commute, right?

Response: Yes, based on a new conceptual framework, the value of the cluster analysis is stated in lines 452-460: ‘**The results from GWR models have implicitly detected a double-edged effect of the built environment, that the impact of a particular variable on home-based and work-based commuting could be contrary at a spatial unit. For example, the work population decreases home-based commuting distances but increases work-based commuting distances in suburban areas. The impact is also spatially heterogeneous. A key question is what the combined effects of all built environment variables are at a particular location. To reveal the double-edged effect explicitly, a two-step cluster model is applied to further examine the area-based impact of the built environment and answer Research Question 3. ‘**

Other editorial suggestions:

• On page 2, “Most studies of commuting and built environment are based on global models, which assume the impact of built environment is spatially unique.” It should be “spatially homogeneous”.

Response: Yes, we revised it.

• “Human activity in a city is naturally heterogeneous, i.e. the distributions of populations, daily activities and traffic flows.” the writing needs to be revised to focus on the heterogeneity of the relationships (instead of human activity).

Response: Yes, we revised it as **‘However, their relations in a city are naturally heterogeneous, causing spatial issues [7] because of locational effects.’**

• On page 3, “[13] investigated the relation of VMT [vehicle miles traveled] and jobs-housing ratio.” Double check the citation requirement of the journal. Normally, the paper still needs to list the authors’ names in citations.

Response: We have revised them according to the citation format requirement.

• On page 5, “to reveal the heterogeneous effect explicitly, it is necessary to apply a local model, a geographically weighted regression model e.g., to give an overview of the structural relation between commuting and built environment of the whole city”. Move “e.g.” before “a geographically weighted regression model”.

Response: Yes we moved it.

1. Guangzhou Statistics Bureau: http://tjj.gz.gov.cn/tjdt/content/post_5727607.html
