# Supplementary material for: The spatially heterogeneous and double-edged effect of the built environment on commuting distance: Home-based and work-based perspectives
Source: PLoS One. 2022 Mar 23;17(3):e0262727. doi: 10.1371/journal.pone.0262727 (PMC8942273; doi:10.1371/journal.pone.0262727)
Supplement: Attachment — Submitted filename: response-ok.docx [file pone.0262727.s003.docx]

GENERAL COMMENTS TO THE EDITOR AND REVIEWERS:

We are pleased to resubmit our revised manuscript of PONE-D-20-19283, which entitles ‘Spatially heterogeneous and double-edged effect of built environments on the commuting distance: from home-based and work-based perspectives’. We appreciate thoughtful comments and constructive suggestions of the reviewers.

We explain how we revised the paper based on each comment as outlined below.

RESPONSE TO EDITOR:

1. There are lots of language issues, still. For example, “rich literatures”, “commuting distances”, “built environment’s impact”, “considering…, conclusions”, Two “however in Line 57”, and so on. Please do find a native proofreading editor to go through the whole manuscript.

Response: Thanks for your careful work. Yes, we have invited an native editor to do the proofreading through the manuscript.

2. Similarly, some statements are vague and confusing. Like the paper title, which features of commuting do you focus on? Volume of commuters? Commuting means a lot, it could be distance, time, volume or more. Be specific.

In the abstract, “the analysis generally assumes…”, such a statement is not academically rigorous, because it is not a “general” truth in built environment-travel studies. “To fill in the research gaps…” what gaps? It needs to be specific. Also, what are “home-based” and “work-based perspective”? They may be explained in the manuscript, but it confuses the readers without much context in the Abstract. I cannot point out all the academic writing issues here, but you should rewrite some sentences in the whole manuscript to make each sentence clear and connected with context.
Response: We changed the title as ‘Spatially heterogeneous and double-edged effect of built environments on the commuting distance: from home-based and work-based perspectives’.

Response: We changed the title as ‘*Spatially heterogeneous and double-edged effect of built environments on the commuting distance: from home-based and work-based perspectives*’.

We revised the abstract part as: ‘*Linear models assume that the influence of the built environment is spatially homogeneous. However, given the spatial heterogeneity of urban space, conclusions might be different or even be contrary. The influence of the built environment might also be different by home and work locations. To explore the spatially heterogeneous effect of the built environment from both home-based and work-based perspectives, this study applied large-scale cellular cellphone data in Guangzhou, China.*’

3. As mentioned by one reviewer, the definition of home-based and work-based commuters are weird. Each commuter is both a resident and an employee. So how can we say “residents and employees” are “two distinct groups of commuter” (Line 80)? Are they just similar to the concepts of Trip attraction and trip departure in traditional transport literature.

Now I see your definition of “commuting” as “decay parameters of probabilistic distributions of commuting distances” what is P,D in Equation (1), how beta is estimated is unclear, some examples to interpret beta could be helpful. Do you need to standardize the data since you use an estimated parameter to represent commuting. So another issue here is how to define beta as what kind of commuting feature in your study. You can’t generally say it as “commuting”, as pointed out in Comment #2. Why not defining clearly what beta means, then using the a very clear concept to replace the vague statement of “commuting”. If I don’t misunderstand your definition, you actually look at the probability of long-distance commuting departing versus arriving at a zone (spatial cell), right? Is that what the “home-based” versus “work-based” commuting represents?

Response: Thanks for your comments. The definition of home-based and work-based commuting is determined how the commuting distance is aggregated into a spatial unit. In aggregate analysis, the commuting distance of a spatial unit is generally measured by the average value of all travelers’ commuting distances within that unit. It is important to note that, for the same spatial unit or neighborhood, there are two ways of averaging the commuting distance: home-based measure and work-based measure. The former measures the average travel distance of commuters who depart from the spatial unit, while the latter is based on commuters who arrive at the spatial unit. Because of the results of two measurements are different, it is necessary to differentiate between the home-based and work-based commuting distances. Current studies are limited in not considering the relations from home-based and work-based perspectives simultaneously. In addition, this study used the concept of ‘distance decay parameter’ instead of the average distance.

We replaced D by X in the equation (1) to make it consistent with a general form of CDF: P(X<=x). Beta is estimated by a maximum likelihood method. The likelihood is given by eq.2.

The concept of beta is explained as follows (lines 353-361): *beta is estimated by maximizing L(beta,x) and we get: beta=mean(x). It implies that the estimated beta value equals the mean of observed samples of commuting distances. Despite that, measuring the commuting distance by a decay parameter cannot be simply seen as averaging all commuting distances in a spatial unit. The decay parameter beta represents the travel distance distribution of all commuters. For example, small beta value indicates a higher probability of shorter commuting distance and lower probability of longer commuting distance, whilst large beta value indicates a higher probability of longer commuting distance and lower probability of shorter commuting distance. The decay parameter also represents the expectation of the distance distribution in an exponential distribution. beta is not simply a mean of commuting distances. Rather, we should treat it as a weighted average of that, according to the property of an exponential distribution. In other words, the expectation value also shapes the slope of the curve. It represents either a high probability of longer distances and a low probability of shorter distances (large beta), or a high probability of shorter distances and a low probability of longer distances (small beta).*

4. Related to the conceptualization of heterogeneity, or the “contextual effect”, you may refer to Zhang and Zhang 2018a,b in JPER and Urban Studies.

Response: Thank you for providing these critical references. We added them in the introduction part (lines 58-62): *‘Among these reasons, the spatial context is the most important one since it has both direct and indirect effects on travel. The direct effect means that built environment is treated as one of influencing factors along with transportation services and social demographic factors, such as in linear models. The indirect or moderating effects include multiplicity, interaction and scalability [7,8].’
7.	Zhang M, Zhang W. When Context Meets Self-Selection: The Built Environment–Travel Connection Revisited. J Plan Educ Res. 2020;40: 304–319.
8.	Zhang W, Zhang M. Incorporating land use and pricing policies for reducing car dependence: Analytical framework and empirical evidence. Urban Stud. 2018;55: 3012–3033.*

RESPONSE TO REVIEWER #3:

1.According to reponse" home-based commuters who depart from the spatial unit,
while the latter is work-based commuters who arrive at the spatial unit", Usually a person starts from space unit A(home) to space B(work) , there is a problem: The same travel behavior is not only the starting point of space a, but also the arrival point of space B, should it be counted twice as both home and work based commuting?Another problem is that he work and resident at same space unit.How to Identify and differentiated two groups of commuters – residents and employees through the data processing, please give processing flow and why did you do that.

Response: Thanks for your careful review work. In aggregate analysis, the commuting distance of a spatial unit is generally measured by the average value of all travelers’ commuting distances within that unit. It is important to note that, for the same spatial unit or neighborhood, there are two ways of averaging the commuting distance: home-based measure and work-based measure. The former measures the average travel distance of commuters who depart from the spatial unit, while the latter is based on commuters who arrive at the spatial unit. Because of the results of two measurements are different, it is necessary to differentiate between the home-based and work-based commuting distances. Please check the statement in lines 87-95.

2.The independent variables need clarifications. How are dependent variables indicators?
Response: Yes, we added a description of independent and dependent variables in details in the section ‘Variables’. The dependent variables are the decay parameter beta in equation P(X<=x)=1-exp(-x/beta) (x>=0). It implies that the estimated beta value equals the expectation of the distance distribution in an exponential distribution. beta cannot be simply seen as averaging all commuting distances in a spatial unit. Rather, we should treat it as a weighted average of that, according to the property of an exponential distribution. In other words, the expectation value also shapes the slope of the curve. It represents either a high probability of longer distances and a low probability of shorter distances (large beta), or a high probability of shorter distances and a low probability of longer distances (small beta).

3.As the REVIEWER #2 suggest"Also, need to add a conceptual framework explaining why the relationships could be spatially varied". The figure added is so simple and cannont explain, more detailed influence action path and mechanism are needed.

Response: Thanks for your suggestion. We remade the conceptual framework (figure 1).

RESPONSE TO REVIEWER #4:

This is an interesting study, and I think the authors' data material and their findings of differentiated relation between land use and commuting in 4 different clusters can potentially result in a published article of high importance. However, in its present form, the paper has some serious shortcomings that must be amended before I can recommend publication.

1. For one thing, the author uses two ambiguous concepts (i.e. home-based commuting, work-based commuting). Of course, I think I understand what the meaning is here; each spatial unit may become a residential area or workplace area, depending on the direction of commuting. But why not directly use the residential area and workplace area here, or you can define a spatial unit to have both residential area and workplace area attributes. In short, ‘home-based commuting’ and ‘work-based commuting’ are not general academic terms.

Response: Thanks for your careful review work. In aggregate analysis, the commuting distance of a spatial unit is generally measured by the average value of all travelers’ commuting distances within that unit. It is important to note that, for the same spatial unit or neighborhood, there are two ways of averaging the commuting distance: home-based measure and work-based measure. The former measures the average travel distance of commuters who depart from the spatial unit, while the latter is based on commuters who arrive at the spatial unit. Because of the results of two measurements are different, it is necessary to differentiate between the home-based and work-based commuting distances. Please check the statement in lines 87-95.

2. In Literature review section, the author needs to add some literature about the relation between built environment of residential area and workplace area, and the commuting. You can easily search for them on the google scholar. This is the most relevant research to your research. Further, you may find that your research question is not a huge gap.

Response: Yes, we added a new literature review part about the relation between built environment and commuting distances (lines 139-201).

3. In the framework, the author does not control any socioeconomic attributes, which may cause inaccurate results. For aggregated data, both statistical yearbooks and censuses can provide basic socioeconomic attributes in Chinese city, such as GDP, gender ratio, and elderly population ratio. Second, this research used a “5Ds” framework, but ignored Design and Distance to transit. Why? Because public transit accessibility, such as distance to bus/subway station or density of bus/subway station, is generally considered to be a very important factor affecting commuting. I think the author needs a very convincing reason here or needs to re-examine the framework.

Response: We did not include the socioeconomic attributes in the model. Our reason is that ‘*This study does not include socioeconomic attributes, such as GDP, gender ratio, or elderly population ratio. The aim of this analysis is to explore the relation between the built environment and the commuting distance. By revealing the relation, the government can directly implement planning and policy measures for the built environment to address the problem of long commuting distances.*’ (lines 382-386)

Thanks for your suggestion about Design and Distance to transit. We added two new variables: road intersections and bus stops. Based on that, we did new analysis using GWR and two-steps cluster models.

4. For Variable Section, functional facility density belongs to Diversity rather than the Density (Pg. 15, line 295, 304-306; Ewing and Cervero 2010). Second, why choose Zhujiang New Town as the city center, and is Guangzhou a monocentric city? (Pg. 17, line 332-334). However, based on your statement: “The provision of housing encourages employees in the university to live near their workplaces, so the jobs-housing would be balanced. The work population decreases commuting distance in suburban areas (Pg. 22, line 413-415)”. In traditional monocentric city research, home-work distance is positively correlated with the distance to the city center. If you find that there is a low commuting distance area in the suburbs of Guangzhou, then I think Guangzhou may not be a monocentric city. So, you may need to re-examine how many city centers there are in Guangzhou. Besides, Closeness is not a general built environment variable; why use Closeness rather than road network distance to city center.

Response: According to the definition of density, it refers to ‘the variable of interest per unit of area (Ewing and Cervero, 2010)’. We think a single functional facility’s number still belongs to density, because it is measured by each type separately. We also have a diversity measurement, which calculates the POI mixture of all types.

Based on our previous analysis, we found that Guangzhou is a mono-centric city, and the center is Zhujiang New Town (Zheng et al. 2021). Although the work population decreases commuting distance in suburban areas, the city is still a monocentric city.

Closeness is actually a measurement of the centrality of the road network. A road with the highest closeness means that it is close to all roads in the study area, and it is the geometry center of road networks. We do not use the road network distance to center, because it is too similar with the distance to city center variable.

5. Method section lacks an introduction to Two-step cluster models.
Response: Thanks for your suggestion. We added an introduction of the two-step cluster model (lines 467-474): ‘*To reveal the double-edged effect explicitly, a two-step cluster model is applied to further examine the area-based impact of the built environment and answer Research Question 3. The algorithm of the two-step cluster can be referred to Rundle-Thiele et al. [48]. In brief, the first step pre-clusters the data into several small sub-clusters by a cluster feature tree, and the second step aggregates sub-clusters into clusters by the standard hierarchical clustering algorithm. The clustering algorithm could generate different number of clusters. The optimal number of clusters is determined by Schwarz’s Bayesian information criterion (BIC)*.’

6. I suggest that the author add an introduction map of the research area, may be in the Study area section, otherwise it is difficult for readers to understand where the airport suburb, university town, and Huangpu suburb are.
Response: Thanks for your suggestion. We added a map of the study area (Figure 2).

7. Pg. 3, line 55: the authors write: "Among these reasons, the spatial context is the most important one". Why? You need some evidence.
Response: The spatial context is import because it is a scientific issue which is not fully discussed in literature. Issues of built environment measurement and data source are relatively less important than the spatial context since they are method and technical issues which can be solved by improving the model. Studies from Zhang and Zhang (2018, 2020) confirm the opinion. We state it in the texts that ‘*Among these reasons, the spatial context is the most important one since it has both direct and indirect effects on travel. The direct effect means that built environment is treated as one of influencing factors along with transportation services and social demographic factors, such as in linear models. The indirect or moderating effects include multiplicity, interaction and scalability [7,8]. These components cannot be directly measured by linear components.*’ (lines 58-63)

8. Pg. 5, line 86: the authors write: "Home-based commuting represents the morning traffic peak and work-based commuting represents the evening traffic peak". I don’t agree, because commuting is two-way. Both of residential area and workplace area are very congested in the morning and evening.
Response: Yes, we realize your comment is correct. We remove the statement.

9. Pg. 5, line 98: "Area-based planning strategies" is an ambiguous scale. You need a clear definition.
Response: We revised it into ‘zonal planning’.

10. Pg. 13, line 256-260: You can merge this paragraph into the following paragraphs about land use variables (Pg. 16, line 317-325).
Response: Yes, we merged them. Thanks for your suggestion.

11. I would also like to see more explicit acknowledgement that this is a cross-sectional that reveals associations but does not prove causation. The authors should be qualified in their language, and they should be careful to use wording that does not suggest a longitudinal study or causal relationships.
Response: Yes, this is a cross sectional study that reveals the associations.

12. Other minor comments/typo/errors
Pg. 4, line 57: A typo: one 'However' should be deleted
Response: Yes, we deleted one.

Pg.12, line 237: the authors write: " Guangzhou is the largest city in Southern China and Provincial Capital". This is ambiguous statement. How to define the largest city? Area, population, or GDP? How about Shenzhen and Hongkong?
Response: We changed the statement as ‘*the study area is the inner city of Guangzhou, China. Guangzhou is one of the four first-tier cities in China and Provincial Capital.*’

Pg. 13, line 250 and 252: “it defines a residential location” is ambiguous. This is a Chinese expression.
Response: We changed it as ‘the location is defined as his/her residential place’.
Pg. 13, line 259: “27349” -> “27, 349”
Response: Yes.

Pg. 13, line 266-267: in equation one, you need give a definition for ‘D’?
Response: We re-edit the equation using x and X instead of d and D. P(X<=x) is the probability (CDF) that the travel distance X is less than value x, since the probability of a continuous random variable can be only expressed by a cumulative distribution function.

Pg. 15, line 299-300: ‘[14]’ -> ‘Rivera and Tiglao (2005)’
Response: Yes.

Pg. 18, line 346-347: “there is no need to measure the built environment from adjacent cells again” why? Do you think the surrounding environment of adjacent cells will not affect the commuting of travelers?

Response: Travelers’ commuting is already affected by adjacent cells in a GWR model. Thus, for the variables themselves, there is no need to consider adjacent cells again. Otherwise it conflicts with the GWR model.

Pg. 19, line 366: ‘bandwidth’ needs further explanation
Response: Bandwidth is the search range of the model. A cell is affected by all other cells within the range of the bandwidth. Bandwidth can be manually chosen or determined by criteria such as cross-validation or Akaike information criterion (AIC). Please also check it in lines 461-465.

Pg. 19, line 374-375: I know AIC, but what is AICc? Based on your statement: “difference between the two models is more than 3”. Does this mean the difference of AIC value between the two models is greater than 3?
Response: AICc is AIC with a small sample correction. AICc = AIC + 2K(K + 1) / (n - K - 1) where K is the number of parameters and n is the number of observations. When the [sample](https://en.wikipedia.org/wiki/Sample_(statistics)) size is small, there is a substantial probability that AIC will select models that have too many parameters, i.e. that AIC will overfit. To address such potential overfitting, AICc was developed: AICc is AIC with a correction for small sample sizes. Please check it in lines 489-496.

Pg. 20, line 381, 382: This looks like two tables, and the significance should be marked in the table with * or something else.
Response: Yes, we have split it into two tables. Significant variables are shown in maps with color, insignificant results are in grey color.

Pg. 22, line 427: “servicesencourages” -> “services encourages”
Response: Yes.

Pg. 31, line 590-591: “In contrast, mixed development would worsen the traffic in the morning peak.” I think your research results may be difficult to support such a conclusion.

Response: We revised as ‘*In contrast, mixed development is associated with long home-based commuting trips*’.
