# Supplementary material for: The spatially heterogeneous and double-edged effect of the built environment on commuting distance: Home-based and work-based perspectives
Source: PLoS One. 2022 Mar 23;17(3):e0262727. doi: 10.1371/journal.pone.0262727 (PMC8942273; doi:10.1371/journal.pone.0262727)
Supplement: Attachment — Submitted filename: Response to reviewers.docx [file pone.0262727.s004.docx]

RESPONSE TO EDITOR:

Additional Editor Comments (if provided): The revision is significantly improved. Besides the comments from the two reviewers, the authors can further proofread and refine the language, and make it more succinct for readers (e.g., by separating long paragraphs into two and deleting some unnecessary sentences or conjunctions)

Response: Thanks for your suggestion. We made another round of proofreading. We improved the language in several sentences, for example:

| Lines | Before | After |
| --- | --- | --- |
| 53 | It has been argued that the co-location of housing and jobs would not significantly reduce commuting distance [6] and that mixed land uses have no significant impact on commuting distance [7]. | The co-location of housing and jobs would not significantly reduce commuting distance [6]. And mixed land uses have no significant impact on commuting distance [7]. |
| 81 | Hence, researchers should consider the spatially heterogeneous effect to extend the understanding of commuting and built environment relations from a global context to a spatially varied and localized context. | Hence, researchers should consider the spatially heterogeneous effect. It helps extend the understanding of commuting and built environment relations from a global context to a spatially varied and localized context. |
| 87 | The model estimates a set of spatially varying coefficients, which can capture heterogeneous effects. Different from a ‘global’ linear regression model which estimates an averaged single coefficient value across the entire study area, GWR is a ‘local’ model that exhibits complex correlations in different areas. | The model estimates a set of spatially varying coefficients, which can capture heterogeneous effects. It is different from a ‘global’ linear regression model which estimates an averaged single coefficient value across the entire study area. Rather, GWR is a ‘local’ model that exhibits complex correlations in different areas. |
| 112 | In this study, an underlying hypothesis is that the impact of the built environment on home-based and work-based commuting distances may be different or even contrary, causing a double-edged effect. | In this study, an underlying hypothesis is that the impact of the built environment on home-based and work-based commuting distances may be different or even contrary. It causes a double-edged effect. |
| 159 | Levinson [24] used data from 8000 household travel surveys in Washington DC to analyze the accessibility to work and found that residences in job-rich areas and workplaces in housing-rich areas are associated with shorter commutes. | Levinson [24] used data from 8000 household travel surveys in Washington DC to analyze the accessibility to work. It was found that residences in job-rich areas and workplaces in housing-rich areas are associated with shorter commutes. |
| 173 | Geyer and Molayi [26] examined the relationship between average travel time and the job–employed ratio, internal capture ratio and other social-demographic variables and found that workers have higher average travel time in job-rich and balanced areas. | Geyer and Molayi [26] examined the relationship between average travel time and the job–employed ratio, internal capture ratio and other social-demographic variables. They found that workers have higher average travel time in job-rich and balanced areas. |

And there are other revisions on improving the sentences. Please see them in the manuscript with track, at lines 239, 274, 275, 297, 230, 304, 309-310, 313-314, 321-325, 329-332, 348-349, 363, 413, 610-611, 618-619, 629, etc.

RESPONSE TO REVIEWER #3:

1.The logic of introduction is not very smooth，especially line106-112 jump out with other parts, I recommend to put in line 584 “Guidance for area-based planning policies”.

Response: Thanks for your suggestion. We moved the paragraph into line 592.

2.The line 818 “Conclusions and discussions” this part is too thin. Authors should focus on the comparison of the research results with other studies and the reasons for this result.

Response: Thanks for your suggestion. Yes, we added a new part to the conclusions and discussions. We compared our results with other studies. We emphasized the difference part and discussed the reason. The findings of the relationship between the built environment and the commuting distance are different from study to study. The reason is that these studies are based on different theoretical framework. For example, based on the co-location theory and individual choice mechanism, the co-location of jobs and housing reduces the commuting distance. However, other researchers have different opinions. In our study, we gave an explanation. In residential areas where the individual choice is the dominate mechanism, the co-location theory is convincing, but in business centers where the market mechanism is dominating, the co-location of jobs and housing has little effect on the home-based commuting distance. Industrial firms have much stronger bidding ability than individuals. Thus, individuals do not have free choices of housing locations at the business center. Similarly, it also explains why mix development increases the home-based commuting distance. Please see the lines 701–746 for details.

3.As the REVIEWER #2 suggest "Also, need to add a conceptual framework explaining why the relationships could be spatially varied". The figure added is so simple and cannont explain, more detailed influence action path and mechanism are needed. Sad to say that nothing seems to have changed in the revised version.

Response: We appreciate this suggestion very much. After re-considering the theory behind our research findings, we made a new conceptual framework:*‘It assumes that there are two mechanisms which dominate the relationship between the built environment and the commuting distance: the market mechanism and the individual choice mechanism. The relationships are varied at different urban locations, causing spatially heterogeneous effect. Also, the market mechanism and the individual choice mechanism are the leading force of the work-based and home-based commuting, respectively.’* (lines 126-130)

4.Why such hypothesis is puts forward? From the current version, it just lists the achievements of others, rather than discuss the how built environment impact commuting distance.

Response: Thanks for your comment. This question is related to the conceptual framework. We added new statement about the hypothesis underlying the spatially heterogeneous effect: ‘*The debate on the impact of the built environment is mainly caused by the different mechanisms of human and urban space interaction: the market mechanism and the individual choice mechanism. The market mechanism triggers agglomeration economic and shapes the urban spatial structure. Economic agglomeration refers to a large number of firms existing in spatial proximity and benefit from cost reductions and efficiency gains. It encourages capital facilities and buildings to be concentrated located. The individual choice mechanism means a decision maker chooses the residential and work location with the highest utility from a set of alternatives. It assumes that workers choose home locations as close to their jobs as possible. The two mechanisms have different actions on different urban locations. The market mechanism is more competitive at business centers, and the choice mechanism has stronger influence at residential centers and suburban areas. Since the urban space is heterogeneous, the relationship between the commuting distance and the built environment should be spatially varied.’* (lines 56 – 68)

And we also discussed the hypothesis behind the home-based and work-based commuting distance: *‘**Because the results of the two measurements are different, it is necessary to differentiate between home-based and work-based commuting distances. More importantly, the underlying mechanisms are different. From a work-based perspective, the economic agglomeration is the dominant mechanism. Industrial firms have much stronger land-rent bidding ability than individuals in a free market system. It forces local workers to live far from workplaces. From a home-based perspective, the co-location theory is the dominant mechanism. Workers can freely choose their home locations to avoid commuting time cost where the supply of housing land is adequate.’* (lines 99-107)

5.Some other minor defects shows that the author is not serious enough, such as:1)Response: We changed the title as ‘Spatially heterogeneous and double-edged effect of built environments on the commuting distance: from home-based and work-based perspectives’.2)In part of RESPONSE TO EDITOR, response 2 repeated the following paragraph:“Response: We changed the title as ‘Spatially heterogeneous and double-edged effect of built environments on the commuting distance: from home-based and work-based perspectives’.”3)As REVIEWER #4:9response, Area-based planning strategies ere revised into ‘zonal planning”, but I have seen” Guidance for area-based planning policies” as obvious secondary title in line 584.4)Line43 Long commuting “distances” should be “distance”.6. The authors use exactly the same paragraph replied to REVIEWER #3: 1 and REVIEWER #4: 1. Although the two comments are similar to some extent, the authors should still give a targeted answer.

Response: Thank you. This is a very useful suggestion that we learn from your comments. It’s true that the response should be targeted to different question. (for comments 1,2 and 6)

We revised ‘area-based planning’ to ‘zonal planning’ (for comments 3).

We revised ‘distances’ to ‘distance’ (for comments 4).

RESPONSE TO REVIEWER #4:

The authors have made considerable improvements followed up my comments. However, several indicator classification error in the new text should be corrected.

1. In the most land use-travel researches, socioeconomic attributes are often used as the control variables in the model. One important reason is that you need to exclude the potential influence of the difference of socioeconomic attributes on the land use-travel relationships. In other words, if you do not control the socioeconomic attributes, the result can be biased. Therefore, you should at least clarify this limitation in the Discussion.

Response: Thanks for your comments. We discussed the limitation of not incorporating socioeconomic attributes in the new edition: *‘The study is limited in not incorporating individual socioeconomic attributes. Socioeconomic attributes are important factors which affect people’s commuting behavior. It is a common approach to explore the influence of people’s socioeconomic characteristic on the commuting distance, particularly in disaggregate analysis. In this study, we did not consider socioeconomic attributes as independent variables. Our argument is that government can implement spatial planning measures to decrease the commuting distance by improving the built environment, but socioeconomic attributes cannot be easily changed and are not effective policy measures for the government. Nevertheless, socioeconomic attributes still have potential influence on commuting behavior. Excluding them would cause biased results of the built environment and the commuting distance relationship. Realizing the shortcoming, we will incorporate individual-level data to further explore the behavioral drivers of commuting distance in future research.’* (lines 747-759)

2. In 5Ds framework, Density measures the intensity of human activity per areal unit, such as population density and job density, rather than all the variables named density

(Ewing and Cervero 2010). Diversity measures pertain to the number of different land use in a given area and the degree to which they are represented in land area, floor area, or employment (Ewing and Cervero 2010). So, functional facility density is closer to Diversity rather than Density.

Response: Thanks for your comments. We made revisions according to your comment. Please check them in lines 409-422.

3. Besides, space syntax closeness does not seem to belong to Destination accessibility. Destination accessibility measures ease of access to trip attractions, such as distance to employment center, distance to shopping center, and distance to city centers. However, space syntax closeness measures the form of network. So, space syntax closeness looks closer to Design, you may need some literature to support this variable.

Response: In our opinion, Closeness is still a destination accessibility index. Closeness in space syntax refers to the centrality level of road. Closeness (also normalized as syntactic ‘Integration’ (Hiller, 2010)) is a key index of the centrality. It indicates the accessibility and centrality level of spatial units (Li et al., 2019). In other words, it means the closeness of any given road section to all other road sections in the system (Hiller, 1984). Traditionally destination accessibility is measured by the distance to the city center. An underlying problem is that sometimes a city center is arbitrarily selected according to experience and subjective perception of a city. The index of ‘closeness’ solves the problem. It does not predefine a center. Rather, it measures a road section’s overall ease of moving to all other sections. Therefore, a location with higher closeness value has better destination accessibility. We also stated it in the manuscript at lines 426-436.

References:

Hillier B, Stonor T. Space syntax - strategic urban design. City Planning Institute of Japan. 2010.

Li Q, Zhou S, Wen P. The relationship between centrality and land use patterns: Empirical evidence from five Chinese metropolises. Computers, Environment and Urban Systems. 2019;78. doi:10.1016/j.compenvurbsys.2019.101356

Hillier Bill, Hanson Julienne. The social logic of space. Cambridge University Press; 1984.

4. Pg. 21, line 420: Public transit accessibility → Distance to transit

Response: Yes, we revised it according to your suggestion: *‘Distance to transit can be alternatively measured by the number of stations per unit area, namely public transit accessibility. In this study, it is the number of bus stops in a grid.’* (lines 424-425)

5. Please list the corresponding manuscript changes in quotation marks under the reviewer’s questions, if necessary.

Response: This is really a useful suggestion. We list changes in quotation marks to make it easier for reviewing.
